# 

**Published:** 1914-04-04

**Authors:** 


### CONTENTS.

PAGE

**Age Limits for Institutional Posts** ... ... 1

**The Salaries of Poor-Law Medical Officers** ... 2

**Hospital and Institutional News** ... ... ... 3

The Legislation of April 1—Women Doctors and the London County Council—The Matron of Blackpool Hospital Resigns—The Medical Superintendentship at St. George's Hospital—A Leeds Adjunct to Hospital Work—Gift of a Hospital to Orkney—The Ulster Children's Hospital, Belfast—Holidays on the Continent—New Secretary of the Belgrave Hospital for Children—The Renovated Chester Infirmary—The Additions and their Moral—Institute of Pathology at Middlesex Hospital—Limiting the Stay of Sanatoria Patients—The Poor-Law Order's Effect on Salaries—How to Obtain Radium in Poor-Law Infirmaries—Institutional Half-holidays—The Reduction in the Vagrant Population of London—The Shortage of Hospital Accommodation—A Perfected Hospital System—The London School Medical Service—Death after Swallowing Radium—Pharmacists and Radiography—Appeals by Wireless Telegraphy—The Doctors' Wives Association—This Week's Drug Market.

**X-Ray Dosage—A New Radiometer** ... ... 9

The Simplification of Colour-Matching.

**Isolation Hospitals and Scarlet Fever** ... ... 10

The Truth about the Milne Treatment.

[See over.]

### CONTENTS—(*continued*).

| | PAGE |
|---|---|
| **Definitions of Mental Deficiency** ... ... ... | 11 |
| A Criticism of the New Nomenclature. | |
| **The Marriage Law and Mental Disease**... ... | 12 |
| **Swimming in School Therapy** ... ... ... | 13 |
| The Value and Risks of Water Exercise. | |
| **Research and Remedies** ... ... ... ... ... | 14 |
| **Reports on Hospitals of the United Kingdom**— | 15 |
| By SIR HENRY BURDETT, K.C.B., K.C.V.O | |
| Benenden Sanatorium, Kent. | |
| **National Insurance Act Developments** ... ... | 17 |
| The Interim Report of the Fabian Society (*continued*). | |
| **The Doctors' Wives Association** ... ... ... | 18 |
| **The Lloyd Smith Sanatorium Chair** ... ... | 19 |
| A Convenient Method of Moving Patients. | |
| **For Hospital Workers** ... ... ... ... ... | 19 |
| **Round the World** ... ... ... ... ... ... | 20 |
| Fellow-Workers and their Doings. | |
| **The Editor's Letter-Box** ... ... ... ... ... | 21 |
| The Solvency of Approved Societies. | |
| Sanatoria Patients and Dental Benefit. | |

| | PAGE |
|---|---|
| **Doctors' Wills** ... ... ... ... ... ... ... ... | 21 |
| **The Institutional Library** ... ... ... ... ... | 22 |
| School Design and School Treatment—The Problem of Mental Patients—The Prevention of Deafness—Two Volumes on Midwifery—A History of Ambulance Work — Babyhood Literature. | |
| **Appointments** ... ... ... ... ... ... ... | 25 |
| **The River Ambulance Service** ... ... ... | 25 |
| **Personalia** ... ... ... ... ... ... ... | 25 |
| **Gifts and Bequests** ... ... ... ... ... ... | 25 |
| **The National Medical Union (Official)** ... ... | 26 |
| Resolutions on Policy. | |
| **The Institutional Worker** ... ... (Suppl.) | 1 |
| The Filing of Out-patient Case-Papers. | |
| Questions for April. | |
| The Sick and in Need. | |
| Employment and Training. | |
| **Buildings Contemplated** ... ... ... ... ,, | 3 |
| **The Book Market** ... ... ... ... ... ,, | 3 |

